# Development and validation of a nomogram to predict pulmonary function and the presence of chronic obstructive pulmonary disease in a Korean population

**DOI:** 10.1186/s12890-021-01391-z

**Published:** 2021-01-19

**Authors:** Sang Chul Lee, Chansik An, Jongha Yoo, Sungho Park, Donggyo Shin, Chang Hoon Han

**Affiliations:** 1 Division of Pulmonology, Department of Internal Medicine, National Health Insurance Service Ilsan Hospital, Goyang, Republic of Korea; 2 Research Institute, National Health Insurance Service Ilsan Hospital, Goyang, Republic of Korea; 3 Department of Laboratory Medicine, National Health Insurance Service Ilsan Hospital, Goyang, Republic of Korea; 4 Medical Information Management Team, National Health Insurance Service Ilsan Hospital, Goyang, Republic of Korea; 5 Medical Record Service Team, National Health Insurance Service Ilsan Hospital, Goyang, Republic of Korea

**Keywords:** Chronic obstructive pulmonary disease, Machine learning, Spirometry, Primary care

## Abstract

**Background:**

Early suspicion followed by assessing lung function with spirometry could decrease the underdiagnosis of chronic obstructive pulmonary disease (COPD) in primary care. We aimed to develop a nomogram to predict the FEV_1_/FVC ratio and the presence of COPD.

**Methods:**

We retrospectively reviewed the data of 4241 adult patients who underwent spirometry between 2013 and 2019. By linear regression analysis, variables associated with FEV_1_/FVC were identified in the training cohort (n = 2969). Using the variables as predictors, a nomogram was created to predict the FEV_1_/FVC ratio and validated in the test cohort (n = 1272).

**Results:**

Older age (*β* coefficient [95% CI], − 0.153 [− 0.183, − 0.122]), male sex (− 1.904 [− 2.749, − 1.056]), current or past smoking history (− 3.324 [− 4.200, − 2.453]), and the presence of dyspnea (− 2.453 [− 3.612, − 1.291]) or overweight (0.894 [0.191, 1.598]) were significantly associated with the FEV_1_/FVC ratio. In the final testing, the developed nomogram showed a mean absolute error of 8.2% between the predicted and actual FEV_1_/FVC ratios. The overall performance was best when FEV_1_/FVC < 70% was used as a diagnostic criterion for COPD; the sensitivity, specificity, and balanced accuracy were 82.3%, 68.6%, and 75.5%, respectively.

**Conclusion:**

The developed nomogram could be used to identify potential patients at risk of COPD who may need further evaluation, especially in the primary care setting where spirometry is not available.

## Background

Chronic obstructive pulmonary disease (COPD), the third leading cause of mortality worldwide, is a common and preventable disease characterized by progressive airflow obstruction [1, 2]. One of the main challenges in COPD is its frequent underdiagnosis [3]. People with early or undiagnosed COPD have been shown to have significant morbidity from exacerbations many years before their diagnosis, which can burden healthcare costs [4].

One of the most important factors contributing to the delayed diagnosis of COPD is the low use of spirometry in primary care [3, 5, 6]. People with early or undiagnosed COPD are most likely to encounter the healthcare system in the primary care setting. Therefore, earlier diagnosis of COPD in primary care followed by proper management could significantly improve the prognosis [7]. However, primary care providers do not always have access, time, or adequate training to use spirometry for patients suspected of having COPD [8].

Alternatively, an easy-to-use tool to predict spirometry results or the presence of COPD would be helpful in enhancing COPD screening in primary care. When a patient is predicted to have COPD, the primary care provider would refer the patient for spirometry. Previous studies have proposed prediction models for the diagnosis or prognosis of COPD patients [9–16]. However, most of these models require information not available in the primary care setting and were developed mainly in non-Asian populations.

Therefore, the purpose of this study was to develop and validate a history- and symptom-based nomogram that can be conveniently used to predict a spirometry result—the forced expiratory volume-one second (FEV_1_)/forced vital capacity (FVC) ratio—and the presence or absence of COPD.

## Methods

### Study population

**Fig. 1:**
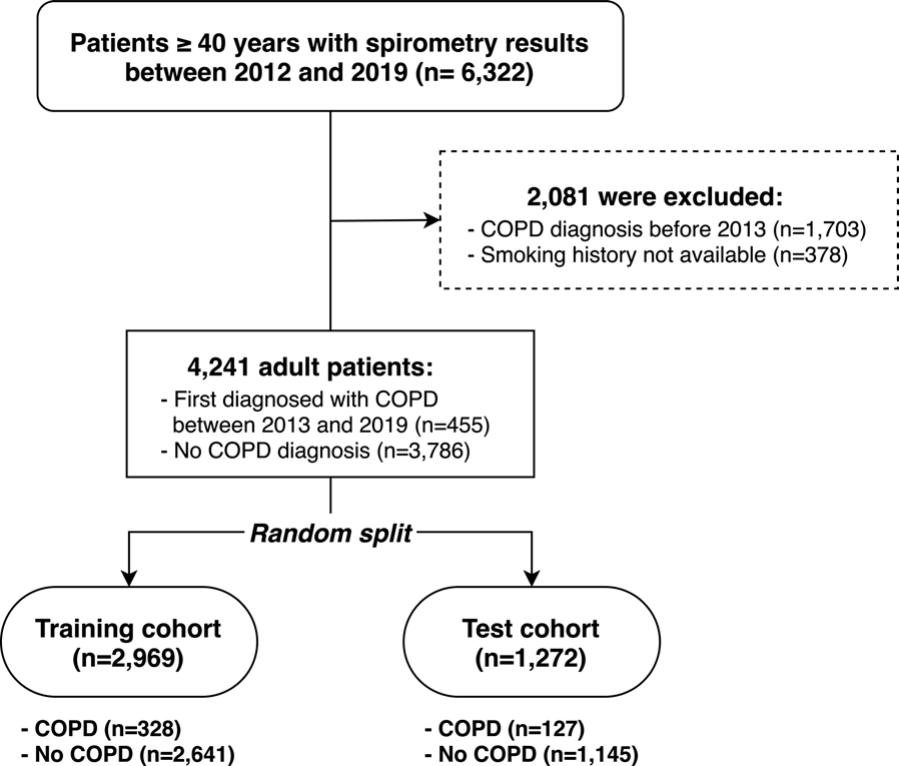
Flowchart of the study population. *COPD* chronic obstructive pulmonary disease

We searched our electronic medical record database and found 6322 adult (≥ 40 years) patients who underwent pulmonary function tests, including spirometry, at a single medical institution in South Korea (hereafter, Korea) between January 2012 and December 2019. Of these, 1,703 patients who were already diagnosed with COPD in 2012 were excluded. Thus, patients included in this study were either first diagnosed with COPD between 2013 and 2019 or were not diagnosed with COPD during the study period. When a patient underwent multiple spirometry measurements, the first test result was used to exclude the possible treatment effect on spirometry results. Of these patients, 378 were excluded because smoking history was missing. Other respiratory ailments, such as asthma, bronchiectasis, or interstitial lung disease, were not included in this study. The final study cohort was randomly split into train and test cohorts with a ratio of 7:3 while preserving the same proportion of COPD patients (Fig. 1). The ratio of 7:3 is commonly used as a rule-of-thumb when splitting a dataset into training and test sets; a recent machine-learning study also reported that using 70% or 80% of the data as a training set showed the best result [17].

### Measurements of lung function and definition of COPD

To assess pulmonary function, spirometry was performed according to the American Thoracic Society/European Respiratory Society (ATS/ERS) standards by trained research assistants [18]. Dry rolling-seal spirometer (Model 2130; SensoMedics, Yorba Linda, CA, USA) was used for all subjects. All spirometry traces were reviewed by a specialist to determine whether they fulfilled the reproducibility and acceptability criteria of the ATS/ERS Task Force.

The normal predictive values for spirometry data were calculated using a reference equation derived from Korea’s general population [19]. A fixed criterion of predicted forced expiratory volume in 1 s per forced vital capacity (i.e., FEV_1_/FVC < 0.7) was used to diagnose patients with COPD in accordance with the Global Initiative for Chronic Obstructive Lung Disease (GOLD) guidelines [20].

### Variables

Outcome variables were the FEV_1_/FVC ratio and the presence or absence of COPD. Predictors for the outcome were age, sex, overweight (defined as body mass index [BMI] > 25 kg/m^2^), smoking history, the symptoms of dyspnea, cough, or sputum, the presence or absence of underlying hypertension, diabetes, congestive heart failure, coronary vascular disease, stroke, or anemia, and the prior use of salbutamol or antibiotics. The information on age, sex, height, weight, and the presence or absence of the symptoms was retrieved from the electronic medical records, while the information on medication and preexisting medical conditions was extracted from the national health insurance claim data. The diagnosis claimed by the healthcare providers and the actual diagnosis may differ because the dataset was established for recording claims and reimbursements. Thus, operational definition was used for determining the presence of medical conditions, as previously reported [21]. For example, hypertension was diagnosed when a patient on antihypertensive medication was admitted for the first time or visited outpatient clinic for a second time with the International Classification of Diseases 10^th^ revision (ICD-10) codes for hypertensive disease. The prior use of salbutamol or antibiotics was defined as the presence of the drug codes with the ICD-10 codes for lower respiratory infections within 3 years prior to the spirometry. Antibiotics included were amoxicillin, clarithromycin, co-amoxiclav, erythromycin, doxycycline and cephalexin.

Smoking history was available from both our medical records and the national health screening results, as all Koreans over 40 years old are mandated to undergo the biannual national health screening, which contains a questionnaire about smoking habits. However, there were approximately 5 times more missing values in the health screening records than the medical records. Therefore, we mainly used smoking history from the medical records; we used the health screening database instead only when smoking history was missing in the medical records.

### Statistical analysis and prediction model

Continuous or categorical variables were compared between the training and test cohorts using *t *test or chi-square tests, respectively. Univariable and multivariable linear regression was performed to determine the association between the risk factors and FEV_1_/FVC ratio and find independent predictors. In the multivariable regression, only variables with a significant association with the FEV_1_/FVC ratio in the univariable regression were used.

A linear regression model for predicting the FEV_1_/FVC ratio was fit in the training cohort and validated in the test cohort using mean absolute error (MAE) as an evaluation metric. In addition, the agreement between the predicted and actual FEV_1_/FVC values was graphically assessed using the Bland–Altman plot. A nomogram to predict FEV_1_/FVC was created based on the prediction model fitted in the training cohort.

Using predicted FEV_1_/FVC values as a diagnostic criterion, the area under receiver operating characteristic curve (AUC), sensitivity, specificity, positive predictive value (PPV), negative predictive value (NPV), and balanced accuracy were calculated for discriminating between patients with and without COPD, with their 95% confidence intervals (CIs).

Our study cohort was imbalanced, with approximately 9 times more patients in the non-COPD group than in the COPD group. In an imbalanced cohort, it is highly likely that predicted outcome values are biased towards the majority group (i.e., non-COPD group or higher FEV_1_/FVC ratio in this study). Therefore, when training the model, we used the synthetic minority over-sampling technique (SMOTE) algorithm to create synthetic minority class cases to balance the two classes [22].

All analyses were performed using R 3.6.0. The packages used include ‘stats (v3.6.0)’ for linear regression, ‘pROC (v1.15.3)’ for AUC analysis, ‘epiR (v1.0–15)’ for calculating diagnostic performances, ‘DMwR (v0.4.1)’ for SMOTE, ‘rms (v5.1–3.1)’ for drawing a nomogram, and ‘BlandAltmanLeh (v0.3.1)’ for drawing a Bland–Altman plot. Two-sided probability values of < 0.05 were considered statistically significant.

## Results

### Patient characteristics

The final study cohort comprised 4241 patients (2204 men and 2037 women) with a mean age of 67 (range, 40–98) years. The mean or frequency of all the variables was not significantly different between the training and test cohorts (Table 1). The prevalence of COPD was 10.7% (455/4241) in the study population. The mean FEV_1_/FVC ratio was 55.3 (standard deviation [SD], 11.3) in the COPD group, and 79.5 (SD, 5.57) in the non-COPD group.

**Table 1:** Patient characteristics

Variables	Training cohort (n = 2969)	Test cohort (n = 1272)	*P* value	Total cohort (n = 4241)
Age (years), mean (SD)	67.3 (12.0)	67 (12.2)	0.493	67.2 (12.0)
Sex, male (vs. female)	1540 (51.8%)	664 (52.1%)	0.858	2204 (51.9%)
Overweight	1147 (38.6%)	475 (37.3%)	0.449	1622 (38.2%)
Smoking	1126 (37.8%)	483 (37.9%)	0.996	1609 (37.9%)
Spirometry results, mean (SD)				
FEV_1_ (L)	2.7 (0.9)	2.7 (0.9)	0.986	2.7 (1.0)
FEV_1_, % pred	97.8 (25.0)	96.9 (24.2)	0.921	97.2 (24.7)
FVC (L)	2.2 (0.7)	2.2. (0.8)	0.901	2.2 (0.8)
FVC, % pred	94.8 (24.1)	94.6 (24.2)	0.919	94.7 (24.2)
FEV_1_/FVC (%)	76.9 (9.8)	76.9 (10.0)	0.904	76.9 (9.9)
FEF_25-75_ (L)	1.6 (1.0)	1.6 (1.0)	0.953	1.6 (1.0)
FEF_25-75_, % pred	56.0 (29.5)	55.6 (31.0)	0.930	55.8 (30.2)
Respiratory symptoms				
Dyspnea	277 (9.3%)	124 (9.7%)	0.708	401 (9.4%)
Cough	300 (10.1%)	123 (9.7%)	0.710	423 (10.0%)
Abnormal sputum	64 (2.2%)	30 (2.4%)	0.765	94 (2.2%)
Comorbidities				
COPD	328 (11%)	127 (10%)	0.334	455 (10.7%)
CHF	66 (2.2%)	30 (2.4%)	0.872	96 (2.3%)
Coronary artery diseases	181 (6.1%)	75 (5.9%)	0.860	256 (6.0%)
Stroke	126 (4.2%)	69 (5.4%)	0.108	195 (4.6%)
Hypertension	1034 (34.8%)	431 (33.8%)	0.585	1465 (34.5%)
Diabetes mellitus	582 (19.6%)	233 (18.3%)	0.355	815 (19.2%)
Anemia	84 (2.8%)	34 (2.7%)	0.858	118 (2.8%)
Acid reflux	266 (8.9%)	100 (7.8%)	0.270	366 (8.6%)
Medication history within 1 year				
One or more salbutamol use	87 (2.9%)	38 (3.0%)	0.997	125 (2.9%)
One or more antibiotics use	83 (2.8%)	43 (3.4%)	0.351	126 (3.0%)

*FEV*_*1*_ forced expiratory volume-one second, *FVC* forced vital capacity, *FEF* forced expiratory flow at 25/50/75 percent of the FVC curve, *COPD* chronic obstructive pulmonary disease, *CHF* congestive heart failure

### Factors associated with FEV_1_/FVC

In the multivariable linear regression analysis, older age (*β* coefficient [95% CI], − 0.153 [− 0.183, − 0.122]), male sex (− 1.904 [− 2.749, − 1.056]), current or past smoking history (− 3.324 [− 4.200, − 2.453]), and the presence of dyspnea (− 2.453 [− 3.612, − 1.291]) were significantly associated with decreased FEV_1_/FVC, while the presence of overweight (0.894 [0.191, 1.598]) was with increased FEV_1_/FVC (Table 2).

**Table 2:** Linear regression results for FEV_1_/FVC ratio in the training set

	Univariable		Multivariable	
	Beta coefficients (95% CI)	*P *value	Beta coefficients (95% CI)	*P* value
Age	− 0.146 (− 0.176, − 0.118)	< 0.001	− 0.153 (− 0.183, − 0.122)	< 0.001
Sex	− 3.863 (− 4.510, − 3.147)	< 0.001	− 1.904 (− 2.749, − 1.056)	< 0.001
Overweight	1.258 (0.534, 1.982)	< 0.001	0.894 (0.191, 1.598)	0.013
Dyspnea	− 2.577 (− 3.772, − 1.370)	< 0.001	− 2.453 (− 3.612, − 1.291)	< 0.001
Cough	1.420 (0.197, 2.644)	0.023	0.400 (− 0.777, 1.576)	0.505
Sputum	− 0.555 (− 2.976, 1.859)	0.652		
Current or past smoking	− 4.075 (− 4.828, − 3.381)	< 0.001	− 3.324 (− 4.200, − 2.453)	< 0.001
Hypertension	− 0.877 (− 1.619, − 0.131)	0.021	− 0.155 (− 0.916, 0.606)	0.690
Coronary vascular disease	− 1.732 (− 3.194, − 0.264)	0.021	0.024 (− 1.415, 1.461)	0.974
Congestive heart failure	− 3.507 (− 5.809, − 1.094)	0.004		
Stroke	− 0.234 (− 1.924, 1.455)	0.786		
Diabetes	− 0.057 (− 0.960, 0.848)	0.903		
Anemia	0.579 (− 1.565, 2.722)	0.594		
Acid reflux	0.524 (− 0.753, 1.800)	0.124		
One or more salbutamol prescription	− 1.995 (− 4.017, 0.023)	0.053		
One or more antibiotics prescription	− 1.523 (− 3.540, 0.504)	0.141		

*CI* confidence interval, *FEV*_*1*_ forced expiratory volume-one second, *FVC* forced vital capacity

### Prediction model

The mean difference between the predicted and actual FEV_1_/FVC ratios (i.e., MAE) was 8.858 in the training cohort and 8.721 in the test cohort. For FEV_1_/FVC in the range between 65 and 75, the MAE was 6.324 in the training cohort and 6.490 in the test cohorts.

In the diagnosis of COPD using the predicted FEV_1_/FVC ratio, the AUC was 0.832 (95% CI 0.812–0.845) and 0.822 (95% CI 0.789–0.854) in the training and test cohorts, respectively. The overall performance was best when the criterion of FEV_1_/FVC < 70 was used to diagnose COPD; the sensitivity, specificity, PPV, NPV, and balanced accuracy were 82.3%, 68.6%, 25.5%, 96.7%, and 75.5%, respectively (Table 3).

**Table 3:** Model performance in discrimination according to the cutoff predicted FEV_1_/FVC ratio in the test set

Cut-off (%)	Sensitivity	Specificity	Precision	NPV	Balanced accuracy
< 65.0	60.5% (52.2–68.5)	83.1% (80.8–85.3)	31.9% (26.5–37.7)	94.2% (92.5–95.5)	71.8% (66.5–76.9)
< 68.0	73.5% (65.6–80.4)	75.4% (72.8–77.9)	28.1% (23.6–32.8)	95.6% (94–96.9)	74.4% (69.2–79.1)
< 70.0	82.3% (75.2–88.1)	68.6% (65.8–71.3)	25.5% (21.7–29.7)	96.7% (95.3–97.9)	75.5% (70.5–79.7)
< 72.0	87.8% (81.3–92.6)	60.6% (57.7–63.5)	22.6% (19.2–26.2)	97.4% (96–98.5)	74.2% (69.5–78)
< 75.0	95.9% (91.3–98.5)	44.9% (420–47.8)	18.5% (15.8–21.5)	98.8% (97.5–99.6)	70.4% (66.6–73.2)

Values in parentheses are 95% confidence intervals

*FEV*_*1*_ forced expiratory volume-one second, *FVC* forced vital capacity, *NPV* negative predictive value

**Fig. 2:**
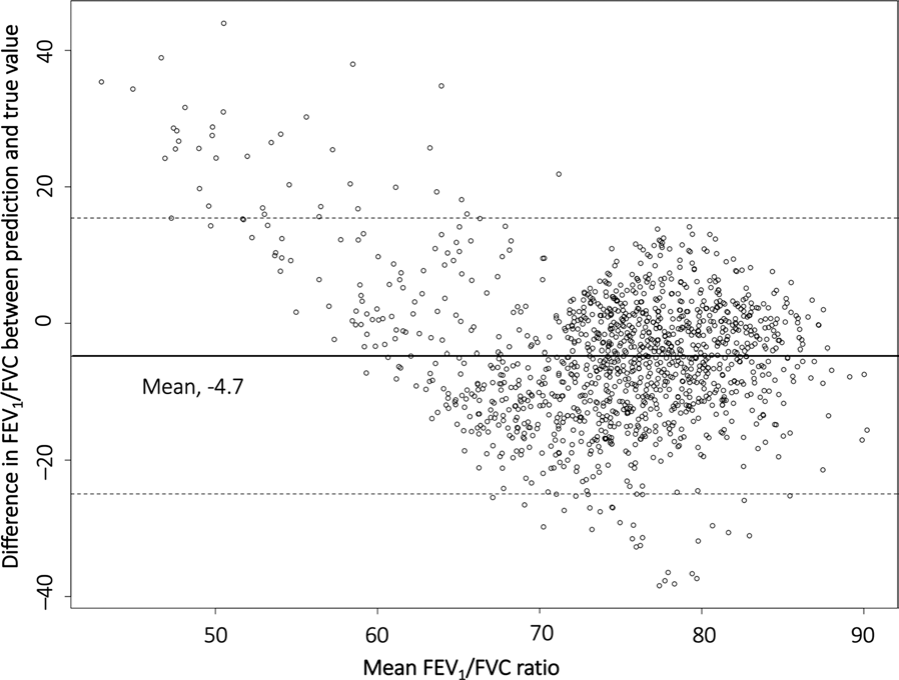
Plot of differences between predicted and actual FEV_1_/FVC ratio versus the mean of the predicted and actual ratios in the test set. The black line represents the mean of the differences, showing the presence of bias, about − 4.7, in the predicted FEV_1_/FVC ratio. The two dotted lines represent the limits of agreement, ± 1.96σ

**Fig. 3:**
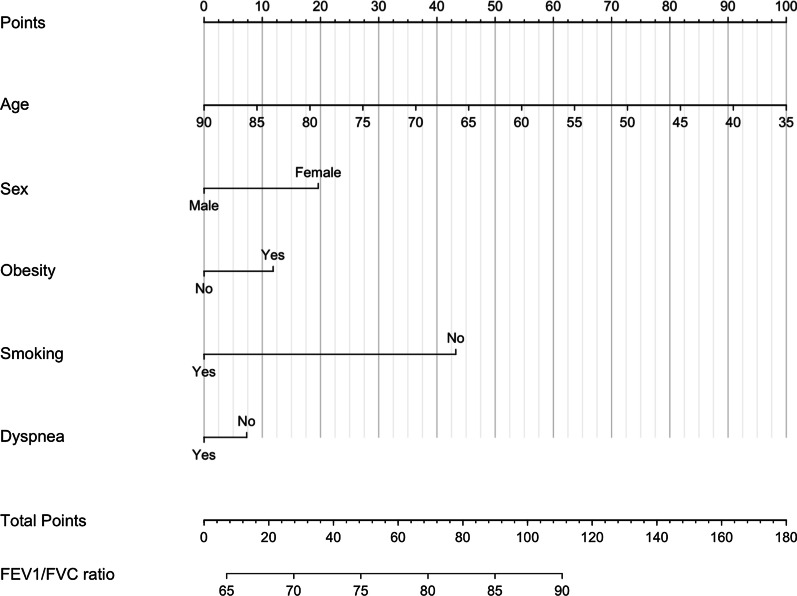
Nomogram predicting FEV_1_/FVC ratio. The nomogram is used by first giving each variable a score on the ‘Points’ scale. The scores for all variables are then added to obtain the total score and a vertical line is drawn from the ‘Total Points’ row to estimate the FEV_1_/FVC ratio. A predicted FEV_1_/FVC value < 65 or > 90 must be interpreted as ‘less than 65’ or ‘larger than 90’, respectively

The Bland–Altman plot revealed a trend that our model overestimated FEV_1_/FVC when an actual FEV_1_/FVC value was less than 65; in this range, many cases were observed above the upper 95% limit of agreement (Fig. 2). Hence, the effective range of the FEV_1_/FVC ratio predicted by our nomogram was from 65 to 90; a predicted FEV_1_/FVC value less than 65 or larger than 90 must be interpreted as ‘less than 65’ or ‘larger than 90’, respectively, instead of the actual value itself (Fig. 3).

## Discussion

In this study, we developed a multivariable model to identify patients who are expected to have decreased pulmonary function and thus is at risk for COPD. In developing this prediction model, we aimed to create an easy-to-use tool that can help primary care providers decide whether to refer patient suspected of having COPD to a facility where spirometry is available. Thus, we examined variables that are obtainable from simple physical examination and history taking for potential predictors.

In our study, the five variables associated with airflow limitation (i.e., decreased FEV_1_/FVC) were older age, male sex, the absence of overweight, the presence of dyspnea, and ever-smoking history. Old age, male sex, and smoking are well-known risk factors for COPD. Historically, COPD has been considered a disease of elderly male smokers, although evidence suggests that this historical view is slowly changing [23]. The prevalence and mortality of COPD have increased more rapidly in women than in men during the past 2 decades, attributed to the changing smoking trends during the past 50 years [24]. Hence, reevaluation of risk stratification by gender is warranted in the future.

Tobacco smoking is the most powerful risk factor for COPD. Although the acquisition of accurate and correct information on the actual smoking habits—duration, amount, and type of cigarette—is of utmost importance, the information in electronic medical records is often quite variable depending on the timing of data entry, visit route (i.e., outpatient, emergency room, or general ward), or medical staff who entered the data. Thus, we processed the smoking data as a binary variable: non-smoker and ever (current or past)-smoker.

In this study, the presence of overweight showed a protective effect, which is in line with previous studies. A study with Asian COPD patients reported that COPD patients with a higher BMI have a better pulmonary function [25]. In another study, while underweight was associated with poor survival in COPD, there was a protective effect of overweight and obesity on mortality on COPD patients [26].

The ATS/ERS guideline recommends using the percentile value rather than fixed percent for interpreting the results of a pulmonary function test because using 0.70 as a lower limit of the FEV1/FVC ratio could yield a significant number of false-positive results in males aged > 40 years and females > 50 years [27]. However, our purpose was to develop an easy-to-use nomogram for primary physicians to decide whether to refer a patient for pulmonary function test. For physicians who are not familiar with spirometry, a fixed percent ratio can be more intuitive and easy-to-read than a percentile value when making such a decision. In addition, in this screening setting, false-negative diagnosis by underestimating the risk is generally considered worse than false-positive diagnosis by overestimating the risk. Furthermore, the results of a pulmonary function test were similar with both the fixed ratio and the percentile value in a previous study [28]. Hence, we used a fixed FEV1/FVC ratio as the outcome in this study.

GOLD guidelines also support the use of multivariable prediction models to assess the prognostic profile and facilitate follow-up of patients, instead of single predictors such as spirometry or history of exacerbations [20]. Several models have been proposed to predict the risk of COPD. However, they either require information that cannot be obtained in the primary care setting [9–11, 13, 15] or were developed for non-Asian populations [9, 10, 13–16]. Therefore, our model may have additional values because it only requires simple physical examination and history taking. In addition, since the occurrence and manifestation of COPD is unique to each race and country [29–32], we believe that our model could screen more undiagnosed COPD patients in Korea. We wish that we could improve our model as more data are obtained in the future and eventually develop a robust, reliable prediction model that can be used nationwide.

This study has some limitations. First, further external validation is needed, because this model was developed with a retrospective study in a single institution. Second, detailed smoking history, such as the type of smoking, amount, and duration, was not used in our analysis. In addition to the conventional tobacco smoking, various electronic cigarettes using new nicotine delivery technologies have recently gained popularity in public. Although recent national health screening questionnaires are changing to reflect recent smoking behavior, the data used in this study did not contain it. Third, we were not able to collect the information on the history of occupation or exposure to chemical irritants, which can also be critical for predicting pulmonary function and the presence of COPD.

## Conclusions

In conclusion, we developed a nomogram to predict an FEV_1_/FVC ratio and the presence of COPD based on age, sex, weight, the presence of dyspnea, and smoking history. This nomogram could be used conveniently to screen potential high-risk patients, especially in the primary care setting where spirometry is not available.

## Data Availability

Due to the institutional policy, data can only be made available to researchers who subject to a non-disclosure agreement, upon reasonable request.

## Abbreviations

COPD: Chronic obstructive pulmonary disease
FEV_1_: Forced expiratory volume-one second
FVC: Forced vital capacity
ATS/ERS: American Thoracic Society/European Respiratory Society
GOLD: Global initiative for chronic obstructive lung disease
BMI: Body mass index
MAE: Mean absolute error
AUC: Area under the curve
PPV: Positive predictive value
NPV: Negative predictive value
CI: Confidence interval
